# Development of Piezo-Driven Compliant Bridge Mechanisms: General Analytical Equations and Optimization of Displacement Amplification

**DOI:** 10.3390/mi8080238

**Published:** 2017-08-03

**Authors:** Huaxian Wei, Bijan Shirinzadeh, Wei Li, Leon Clark, Joshua Pinskier, Yuqiao Wang

**Affiliations:** 1 School of Mechatronic Engineering, China University of Mining and Technology, Xuzhou 221116, China; weihuaxian@yahoo.com (H.W.); cumtwyq@126.com (Y.W.); 2 Robotics and Mechatronics Research Laboratory, Department of Mechanical and Aerospace Engineering, Monash University, Clayton 3800, Australia; bijan.shirinzadeh@monash.edu (B.S.); leon.s.clark@gmail.com (L.C.); joshua.pinskier@monash.edu (J.P.)

**Keywords:** flexure hinge, compliant bridge mechanisms, micro-motion scaling, kinematics

## Abstract

Compliant bridge mechanisms are frequently utilized to scale micrometer order motions of piezoelectric actuators to levels suitable for desired applications. Analytical equations have previously been specifically developed for two configurations of bridge mechanisms: parallel and rhombic type. Based on elastic beam theory, a kinematic analysis of compliant bridge mechanisms in general configurations is presented. General equations of input displacement, output displacement, displacement amplification, input stiffness, output stiffness and stress are presented. Using the established equations, a piezo-driven compliant bridge mechanism has been optimized to maximize displacement amplification. The presented equations were verified using both computational finite element analysis and through experimentation. Finally, comparison with previous studies further validates the versatility and accuracy of the proposed models. The formulations of the new analytical method are simplified and efficient, which help to achieve sufficient estimation and optimization of compliant bridge mechanisms for nano-positioning systems.

## 1. Introduction

In recent decades, piezoelectric actuators (PZTs) have been frequently used in micro/nano-applications including advanced manufacturing, high precision positioning, scanning probe microscopes and biological cell manipulation [1,2,3,4]. The advantages of piezoelectric actuators include precise motion capability, compact size and large blocking force. However, one of their main drawbacks is the relatively small motion stroke, at about 0.1 percent of its length. Consequently, compliant mechanisms are generally employed to scale the displacement in values compatible with PZTs, including bridge [5], Scott-Russell [6], and lever type mechanisms [7].The compliant mechanisms employ flexure hinges instead of rigid joints to eliminate mechanical play and friction, and hence can achieve ultra-precise and smooth motions [8,9]. However, the kinematics of these flexure-based mechanisms is based on the deflections of their flexure hinges, and this has led to techniques for design, analysis and modeling for compliant mechanisms [10,11,12]. 

**Figure 1:**
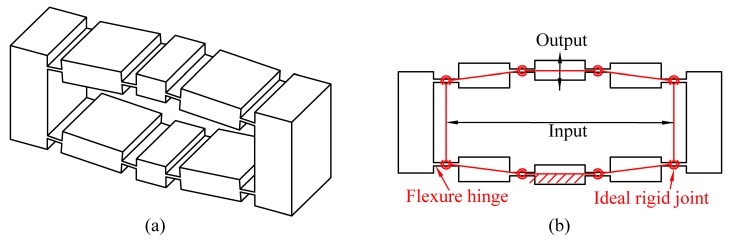
The compliant bridge mechanisms: (**a**) three-dimensional model; (**b**) ideal kinematic model.

Among the commonly used micro-motion scaling mechanisms, the compliant bridge mechanisms, as shown in Figure 1, have been widely used because of their symmetry, compactness and large magnification capability. In the last decade, compliant bridge mechanisms have been widely employed in flexure-based micro-manipulators to provide amplified piezo-actuations [13,14]. With the increasing demands for high-dexterity manipulation, compliant bridge mechanisms have been used as a regular model to construct more complex structures with multi-degrees of freedom [15]. This has led to the requirement for developing an efficient analytical model of displacement amplification for compliant bridge mechanisms.

**Figure 2:**
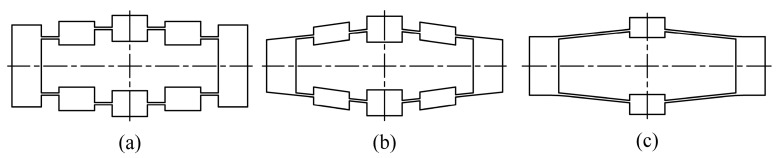
Three types of compliant bridge mechanisms: (**a**) parallel; (**b**) aligned and (**c**) rhombic.

Much research has been directed towards deducing analytical models for compliant bridge mechanisms. Ideal kinematic methods, which treat the flexure hinges as ideal revolute joints, have been shown to be inaccurate, owing to their neglecting elastic deformations in flexure hinges [16,17]. Therefore, an analytical model based on Castigliano’s displacement theory has been developed by Lobontiu [18]. In addition, the matrix method has also been employed as simplified finite element analysis (FEA) [19]. However, the cumbersome formulations of these methods have limited their application. Methods based on elastic beam theory and motion analyses have been used, where analytical equations of displacement amplification and stiffness are obtained [20]. In addition, non-linear models incorporating beam theory of the flexure hinge for high frequencies or large deformation have been developed [21,22]. However, these studies have focused on the analyses of compliant bridge mechanisms that are specifically in parallel [23], aligned [24] and rhombic type [25,26] configurations, as shown in Figure 2. As a result, design processes are separated and repeated for these configurations since the geometric characteristics are not transformable [27,28]. In addition, the design of a compliant bridge mechanism is simultaneously limited by kinematics, stress and stiffness, which are determined by the geometric parameters. Unlike traditional rigid joints, the orientation of the flexure hinge has a significant influence on the mechanism’s performance [29]. For a given application, the optimal design may occur in any of the aforementioned configurations, and hence generalized analytical equations are required for design searches.

The aim in this paper is to investigate a simplified analytical model to be employed within the optimization of displacement amplification for compliant bridge mechanisms covering all types of configuration. In the following section, a method based on beam theory and kinematic analysis is detailed, and analytical equations of input, output, displacement amplification, stiffness and stress are formulated. Subsequently, optimal designs of piezo-driven compliant bridge mechanisms in terms of displacement amplification under kinematic, stress and stiffness constraints have been established. The presented models and optimizations are then verified by FEA and experimental tests. Finally, comparisons of the established models with previous models are carried out, and a theoretic displacement amplification ratio formula of aligned-type compliant bridge mechanisms is attained.

## 2. The General Analytical Model

**Figure 3:**
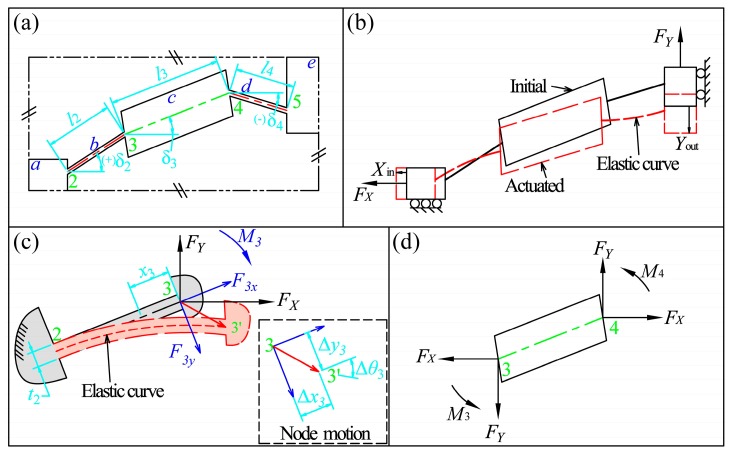
Analytical model of the compliant bridge mechanism: (**a**) general quarter model with configuration parameters; (**b**) schematic of working status; (**c**) deformations of flexure hinge *b*; and (**d**) force equilibrium of the middle link.

As compliant bridge mechanisms generally employ quadrilateral symmetric structures, a general quarter model of the mechanism is analyzed, as shown in Figure 3. The model is composed of five parts: input link *a*, flexure hinge *b*, middle link *c*, flexure hinge *d* and output link *e*. For simplification, four nodes numbered from 2 to 5 are identified between the conjunctions of each part. Six geometric parameters, which are henceforth called *configuration parameters*, are sufficient to determine the configuration of the general compliant bridge mechanisms, as shown in Figure 3a, namely the lengths and orientations of the two flexure hinges and the middle link ($l_{2}$, $l_{3}$, $l_{4}$, $\delta_{2}$, $\delta_{3}$, and $\delta_{4}$). Without loss of generality, the positive directions of orientation angles are defined as shown in Figure 3a, when the central axes of these parts rotate in anticlockwise direction from horizontal position.

The operation can be illustrated by means of the quarter model, as shown in Figure 3b. From the point of view of the mechanics of materials, the flexure hinges deform under the driving forces ($F_{X}$) from the PZT on the input link and the manipulating force ($F_{Y}$) on the output link, and this results in a translational input displacement ($X_{\mathrm{in}}$) and a translational output displacement ($Y_{\mathrm{out}}$) due to the symmetric constraints. The positive directions of the input and output forces and displacements are defined as shown in Figure 3b.

### 2.1. Input and Output Analyses

In order to determine the input and output motions of the compliant bridge mechanism, deflection analyses of flexure hinges are required. Firstly, flexure hinges are analyzed as cantilever beams. Consider the flexure hinge *b*, as shown in Figure 3c, for example, freeing the end (node 3) that is connected to the middle link and let the other end (node 2) be fixed. Using beam theory, the deflections and loads on flexure hinge can be analyzed according to its compliances, that is:

(1) $$\{\begin{array}{c} ∆x_{3}=c_{11}^{b}\cdot F_{3x}\\ ∆y_{3}=c_{22}^{b}\cdot F_{3y}+c_{23}^{b}\cdot M_{3}\\ ∆\theta_{3}=c_{32}^{b}\cdot F_{3y}+c_{33}^{b}\cdot M_{3} \end{array}$$

where $∆x_{3}$, $∆y_{3}$ and $∆\theta_{3}\;$ are the axial deformation, deflection and slope angle of flexure hinge *b* at node 3, respectively. $F_{3x}$, $F_{3y}$ and $M_{3}$ are the axial force, shear force and bending moment, respectively. c is the compliance factor of the flexure hinge which is solely determined by the geometric parameters and material characteristics. For strip-type flexure hinges, the compliances are given as [30]:

(2) $$\{\begin{array}{c} c_{11}^{b}=\frac{l_{2}}{Ewt_{2}}\\ c_{22}^{b}=\frac{4l_{2}^{3}}{Ewt_{2}^{3}}+\frac{l_{2}}{Gwt_{2}}\\ c_{23}^{b}=c_{32}^{b}=\frac{6l_{2}^{2}}{Ewt_{2}^{3}}\\ c_{33}^{b}=\frac{12l_{2}}{Ewt_{2}^{3}} \end{array}$$

where $t_{2}$ is the thickness of flexure hinge, w the width of the mechanism, E the modulus of elasticity, and G the modulus of shear. The axial and shear forces on the free end can be obtained by means of force equilibrium of the mechanism, which can be written as:

(3) $$\{\begin{array}{c} F_{3x}=F_{X}\cdot \cos\delta_{2}+F_{Y}\cdot \sin\delta_{2}\\ F_{3y}=F_{X}\cdot \sin\delta_{2}-F_{Y}\cdot \cos\delta_{2} \end{array}$$

where $\;F_{3x}$ and $F_{3y}$ are the axial and deflecting forces of flexure hinge *b* at node 3, respectively. Similarly, the axial and shear forces of flexure hinge *d* can be obtained as:

(4) $$\{\begin{array}{c} F_{4x}=F_{X}\cdot \cos\delta_{4}+F_{Y}\cdot \sin\delta_{4}\\ F_{4y}=F_{X}\cdot \sin\delta_{4}-F_{Y}\cdot \cos\delta_{4} \end{array}$$

The motion of flexure hinge *d* at node 4 can similarly be identified as: $∆x_{4}$, $∆y_{4}$ and $∆\theta_{4}$. Equations (3) and (4) indicate that the internal loads, and hence the bending moments, within the two flexure hinges are different if they have different orientations. Since the middle link is treated as rigid, the slope angles of the two flexure hinges at node 3 and 4 are always identical. Considering the force equilibrium of the middle link as shown in Figure 3d, an equation system can be established that relates the bending moments of the two flexure hinges, and can be written as:

(5) $$\{\begin{array}{c} F_{3y}\cdot c_{32}^{b}+M_{3}\cdot c_{33}^{b}=F_{4y}\cdot c_{32}^{d}+M_{4}\cdot c_{33}^{d}\\ F_{X}\cdot l_{3}\cdot \sin\delta_{3}=M_{3}+M_{4}+F_{Y}\cdot l_{3}\cdot \cos\delta_{3} \end{array}$$

where $M_{3}$ and $M_{4}$ are the bending moments at node 3 and 4, respectively. By substituting Equations (1)–(4) into Equation (5), the bending moments can be deduced as:

(6) $$\{\begin{array}{c} M_{3}=\frac{F_{Y}\cdot c_{32}^{b}\cdot \cos\delta_{2}-F_{Y}\cdot c_{32}^{d}\cdot \cos\delta_{4}-F_{X}\cdot c_{32}^{b}\cdot \sin\delta_{2}+F_{X}\cdot c_{32}^{d}\cdot \sin\delta_{4}-F_{Y}\cdot c_{33}^{d}\cdot l_{3}\cdot \cos\delta_{3}+F_{X}\cdot c_{33}^{d}\cdot l_{3}\cdot \sin\delta_{3}}{c_{33}^{b}+c_{33}^{d}}\\ M_{4}=\frac{F_{Y}\cdot c_{32}^{d}\cdot \cos\delta_{4}-F_{Y}\cdot c_{32}^{b}\cdot \cos\delta_{2}+F_{X}\cdot c_{32}^{b}\cdot \sin\delta_{2}-F_{X}\cdot c_{32}^{d}\cdot \sin\delta_{4}-F_{Y}\cdot c_{33}^{b}\cdot l_{3}\cdot \cos\delta_{3}+F_{X}\cdot c_{33}^{b}\cdot l_{3}\cdot \sin\delta_{3}}{c_{33}^{b}+c_{33}^{d}} \end{array}$$

Eventually, the translational displacements of input and output links are composed of deflections of the two flexure hinges and the arc motion of the middle link, which can be written as:

(7) $$\{\begin{array}{c} X_{\mathrm{in}}=∆x_{3}\cdot \cos\delta_{2}+∆y_{3}\cdot \sin\delta_{2}+∆x_{4}\cdot \cos\delta_{4}+∆y_{4}\cdot \sin\delta_{4}+∆\theta_{3}\cdot l_{3}\cdot \sin\delta_{3}\\ Y_{\mathrm{out}}=∆y_{3}\cdot \cos\delta_{2}-∆x_{3}\cdot \sin\delta_{2}+∆y_{4}\cdot \cos\delta_{4}-∆x_{4}\cdot \sin\delta_{4}+∆\theta_{3}\cdot l_{3}\cdot \cos\delta_{3} \end{array}$$

By substituting Equation (1) into Equation (7), the closed-form equations of the input and output displacements can be deduced in the form:

(8) $$\{\begin{array}{c} X_{\mathrm{in}}=a_{11}\cdot F_{X}+a_{12}\cdot F_{Y}\\ Y_{\mathrm{out}}=a_{21}\cdot F_{X}+a_{22}\cdot F_{Y} \end{array}$$

where $a_{11}-a_{22}$ are coefficients determined by geometric parameters and material characteristics as detailed in Appendix A. Based on the equation system, the analytical equations of displacement amplification, input and output stiffness can be deduced with simplified formulations.

### 2.2. Displacement Amplification

The displacement amplification is the ratio of the output displacement to the input displacement when the output link is free. Referring to Equation (8), the displacement amplification can be deduced as:

(9) $$da=\frac{a_{21}}{a_{11}}=\frac{\cos\delta_{2}\cdot \left(c_{22}^{b}\cdot \sin\delta_{2}-c_{11}^{b}\cdot \sin\delta_{2}+B\right)+\cos\delta_{4}\cdot \left(c_{22}^{d}\cdot \sin\delta_{4}-c_{11}^{d}\cdot \sin\delta_{4}+A\right)+\frac{l_{3}\cdot \cos\delta_{3}\cdot C}{c_{33}^{b}+c_{33}^{d}}}{\sin\delta_{2}\cdot \left(c_{22}^{b}\cdot \sin\delta_{2}+B\right)+\sin\delta_{4}\cdot \left(c_{22}^{d}\cdot \sin\delta_{4}+A\right)+c_{11}^{b}\cdot \cos^{2}\delta_{2}+c_{11}^{d}\cdot \cos^{2}\delta_{4}+\frac{l_{3}\cdot \sin\delta_{3}\cdot C}{c_{33}^{b}+c_{33}^{d}}}$$

in which $A=\frac{c_{23}^{d}\cdot \left(c_{32}^{b}\cdot \sin\delta_{2}-c_{32}^{d}\cdot \sin\delta_{4}+c_{33}^{b}\cdot l_{3}\cdot \sin\delta_{3}\right)}{c_{33}^{b}+c_{33}^{d}}$, $B=\frac{c_{23}^{b}\cdot \left(c_{32}^{d}\cdot \sin\delta_{4}-c_{32}^{b}\cdot \sin\delta_{2}+c_{33}^{d}\cdot l_{3}\cdot \sin\delta_{3}\right)}{c_{33}^{b}+c_{33}^{d}}$, $C=c_{32}^{b}\cdot c_{33}^{d}\cdot \sin\delta_{2}+c_{32}^{d}\cdot c_{33}^{b}\cdot \sin\delta_{4}+c_{33}^{b}\cdot c_{33}^{d}\cdot l_{3}\cdot \sin\delta_{3}$.

### 2.3. Input and Output Stiffness

The input stiffness of the compliant bridge mechanism is defined as the applied input force corresponding to unit input displacement, whilst the output link is free. Similarly, an equation system can be found as:

(10) $$k_{\mathrm{in}}=\frac{F_{X}}{X_{\mathrm{in}}}=\frac{1}{a_{11}}\;$$

In addition, the output stiffness of the compliant bridge mechanism is defined as the applied output force per unit output of displacement when the input link is free. Consequently, an equation system can be established for the output stiffness:

(11) $$k_{\mathrm{out}}=\frac{F_{Y}}{Y_{\mathrm{out}}}=\frac{1}{a_{22}}\;$$

### 2.4. Stress Analysis

For compliant mechanisms, the maximum motion range is also limited by the maximum stress in the structure. The maximum stress is generated under the maximum loads. Since the positive output force tends to decrease the stress in the flexure hinge, only input force on the input link is taken into consideration, which can be written as:

(12) $$F_{X}^{\max}=F_{\mathrm{PZT}}^{\max}+F_{\mathrm{preload}}$$

where $\;F_{\mathrm{preload}}$ is the preload which is usually essential to eliminate clearance between PZT and the structure. $F_{\mathrm{PZT}}^{\max}$ is the maximum actuating force from the PZT corresponding to the maximum input displacement, by referring to Equation (10), which can be written as:

(13) $$F_{\mathrm{PZT}}^{\max}==X_{\mathrm{PZT}}^{\mathrm{nl}}\cdot k_{\mathrm{in}}$$

where $\;X_{\mathrm{PZT}}^{\mathrm{nl}}$ is the nominal stroke of the PZT. In addition, the true strokes of PTZs are reduced by the compression of the mechanisms, which can be determined as:

(14) $$X_{\mathrm{PZT}}^{\mathrm{tr}}=X_{PZT}^{\mathrm{nl}}\cdot \frac{k_{\mathrm{pzt}}}{k_{\mathrm{in}}+k_{\mathrm{pzt}}}$$

where $\;k_{\mathrm{pzt}}$ is the stiffness of the PZT. The stroke reduction can be neglected when the input stiffness of the mechanism is much smaller than the stiffness of PZT.

Consider again the flexure hinge *b* as an example, as shown in Figure 3c. The flexure hinge can be treated as a cantilever beam under combined loads at the free end. The maximum stress within the flexure hinge is the superposition of the axial and bending stress, which can be written as:

(15) $$\sigma_{23}^{\max}=\underset{x_{3}\in \left[0,l_{2}\right]}{\max}\left(\sigma_{M}+\sigma_{N}\right)$$

where $\sigma_{N}=\frac{F_{3x}}{w\cdot t_{2}}$ and $\sigma_{M}=\frac{6\cdot M_{23}}{w\cdot t_{2}^{2}}$ are the axial stress and maximum bending stress of a cross-section within flexure hinge *b* at the position of $x_{3}$ with respect to node 3, respectively. For a general compliant bridge mechanism, the bending moment varies along the flexure hinge because of the hinge orientation. The moment can be deduced as:

(16) $$M_{23}=M_{3}^{\max}+F_{3y}^{\max}\cdot x_{3},\left(x_{3}\in \left[0,l_{2}\right]\right)$$

where $\;M_{3}^{\max}$ and $F_{3y}^{\max}$ are the maximum bending moment and shear force obtained by Equations (3)–(6) under the maximum input force of Equation (12). Similarly, the maximum stress within the flexure hinge *d* can be obtained as $\sigma_{45}^{\max}$. The maximum stress in the compliant bridge mechanism can be determined as:

(17) $$\sigma^{\max}=\max\;\left(\sigma_{23}^{\max},\sigma_{45}^{\max}\right)$$

## 3. Optimization

Using the established equations, piezo-driven compliant bridge mechanisms can be optimized for maximum displacement under geometric, stress, and stiffness constraints. Herein, a compliant bridge mechanism is optimized for use in a multiple degree of freedom positioner. Eight geometric parameters were investigated as variables, as listed in Table 1. The width of the mechanism was fixed at w=10 mm. Aluminum alloy 7075-T6 was selected as the material with modulus of elasticity  E=72 GPa, a Poisson’s ratio of  μ=0.33, and modulus of shear obtained by $G=\frac{E}{2\left(1+\mu \right)}$.

During the optimization, the contours of the mechanism were constrained by:

(18) $$\{\begin{array}{c} 0.0075\;m\leq l_{2}\cos\delta_{2}+l_{3}\cos\delta_{3}+l_{4}\cos\delta_{4}\leq 0.012\;m\\ -0.01\;m\leq l_{2}\sin\delta_{2}+l_{3}\sin\delta_{3}+l_{4}\sin\delta_{4}\leq 0.01\;m \end{array}$$

The maximum stress is limited by:

(19) $$\sigma_{\max}\leq \frac{\sigma_{u}}{3}$$

where $\;\sigma_{u}=505\;\mathrm{MPa}$ is the ultimate strength of the material. In addition, a nominal actuation of 17.4 µm of the PZT and a preload of 40 N were employed. The input stiffness and output stiffness were constrained as:

(20) $$\{\begin{array}{c} K_{\mathrm{in}}\leq 7\times 10^{6}\;N/m\\ K_{\mathrm{out}}\geq 3.8\times 10^{4}\;N/m \end{array}$$

The objective function is specified by:

(21) Find max:|da|

It can be predicted from Equation (9) that the optimization problem may have many local optima due to the underlying nonlinearity of the model. Therefore, instead of deriving a specific optimization method, a vast quantity of optimizations was carried out using the constrained nonlinear multivariable optimization function “fmincon” in MATLAB (R2013a, MathWorks, Natick, MA, USA) in this study. In each instance, the objective function, boundaries and constraints were the same as stated previously, whilst a random initial estimate within the parameter ranges was used.

### 3.1. Optimization Results

**Figure 4:**
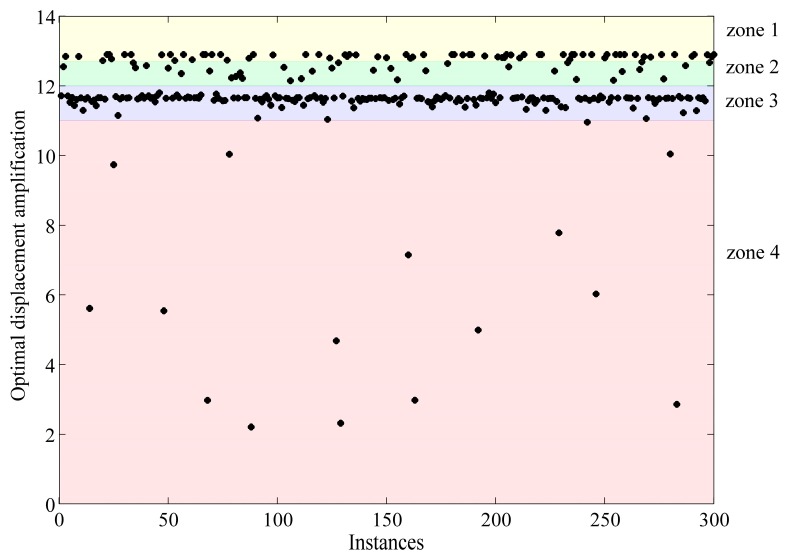
Distribution of the optimal displacement amplifications of the 300 instances with random initial estimations.

**Figure 5:**
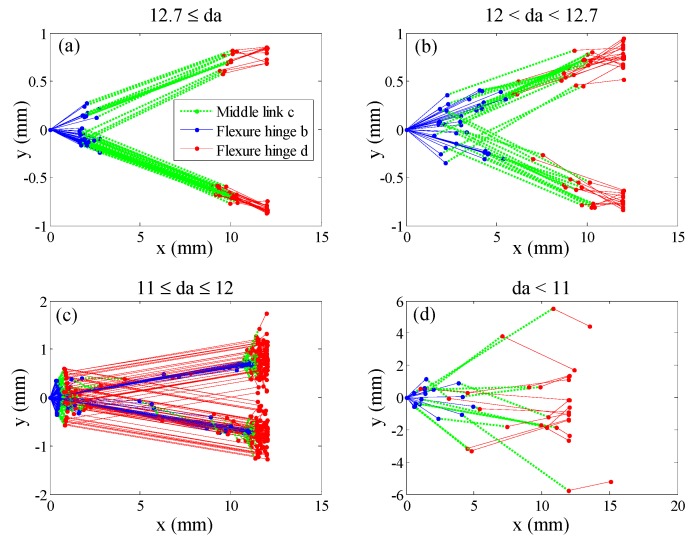
Illustrations of the configurations, zoned according to the values of the optimal displacement amplifications of the 300 instances with random initial estimations: (**a**) 12.7 ≤ *da*; (**b**) 12 < *da* < 12.7; (**c**) 11 ≤ *da* ≤ 12; (**d**) *da* < 11.

As shown in Figure 4, after using 300 solving instances with random initial estimates, the global maximum displacement amplification obtained by the optimization was around 12.8. In addition, various local optima were obtained which are greatly influenced by the initial estimates. The distributions of all the optima can be divided into four zones, as shown in Figure 4, where the quantity of instance from top to down are 70, 33, 181 and 16. The configuration of each instance is illustrated by plotting the central axis of the two flexure hinges and the middle link, as shown in Figure 5, where the origin of the coordinate system is set at node 2, with the *x* axis reverse to the input direction and *y* axis along the output direction. As can be seen, most samples in zone 1 are in aligned configurations, whilst most samples in zone 3 are in rhombic configurations. The optimal design in terms of displacement amplification under the constraints in this study is in the aligned configuration, and the optimal geometric parameters are determined as shown in Table 1.

## 4. FEA and Experimental Evaluations

### 4.1. FEA

**Figure 6:**
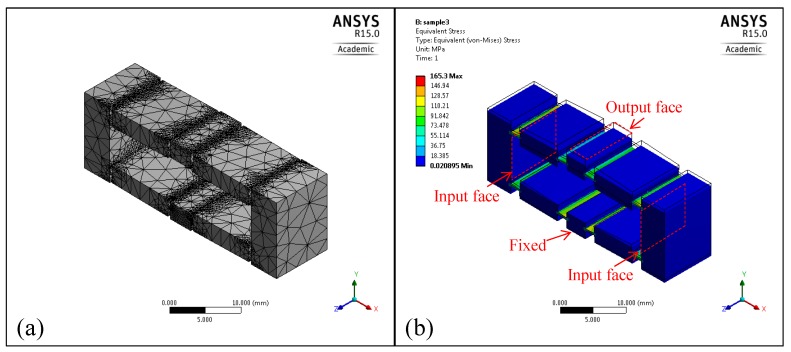
Finite element analysis of the global optimal compliant bridge mechanism: (**a**) mesh model; (**b**) maximum stress simulation.

To verify the models and optimization, the mechanism obtained in previous section was further investigated using FEA and experiment. The model of the whole compliant bridge mechanism was constructed and analyzed within the ANSYS software package (15.0.7, ANSYS, Canonsburg, PA, USA). As shown in Figure 6a, a mesh model with 83,667 nodes and 42,955 elements was built, with refined mesh on the flexure parts. During the analyses, the bottom face of the mechanism was fixed, and a translational input force of 10 N is applied to the two input faces. The average displacements of the input and output faces were recorded, as shown in Table 2. The displacement amplification and input stiffness were then obtained. According to the input stiffness calculated by FEA, an input force of 152 N was actuated on the input faces to simulate the maximum PZT actuation of 17.4 µm with the preload of 40 N. The stress in such a situation was recorded as shown in Figure 6b. Then, in order to investigate the output stiffness, the output face was actuated by 10 N, while the input faces remained free. The results indicate that the deviations between the FEA and the analytical results are less than 11%.

### 4.2. Experimental Evaluation

**Figure 7:**
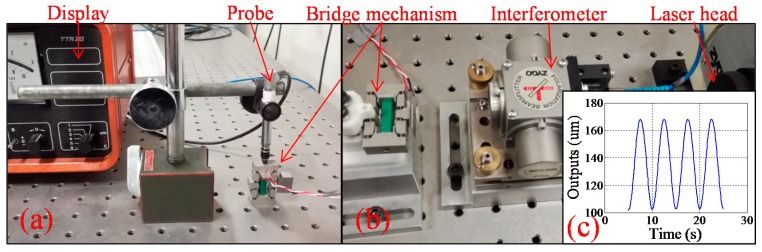
Photos of experimental apparatus: (**a**) setup of input stroke test; (**b**) setup of output displacement test; (**c**) outputs of sinusoidal actuations.

A prototype of the optimal compliant bridge mechanism was fabricated and tested, as shown in Figure 7. The prototype was manufactured from a piece of aluminum alloy 7075-T6 by wire-electrical discharging machining. A PZT (AE0505D16F, NEC, Tokyo, Japan) was inserted into the bridge mechanism and actuated by a controller (MDT693B, Thorlabs, Newton, NJ, USA). During the tests, the PZT was physically preloaded by two identical wedges which are placed together between the actuator and the input link of the compliant bridge mechanism. The PZT was adjusted and fastened manually, where the actuator could efficiently drive the input links. To ensure a constant actuation force during the experiments, the input stoke and the output displacements were tested under the same setting of preload. In the test of the input stroke, as shown in Figure 7a, one of the input links was fixed on the vibration-isolated table while the displacement of the other input link was measured by a position measuring probe (32.10924, TESA, North Kingstown, RI, USA) and read out by an analogue display (TTA20, TESA).The maximum input displacement measured was 13.5 µm. Then, the output displacement of the mechanism was tested as shown in Figure 7b, where the bottom face was mounted and the output displacements were measured by a laser interferometer (7003A, ZYGO, Berwyn, PA, USA). As shown in Figure 7c, the output displacement under the sinusoidal actuation was recorded and the detected maximum output displacement is 168 µm. As shown in Table 3, the analytical displacement amplification for the developed compliant bridge mechanism deviates less than 4% from the experimental result, and 8% with respect to the FEA result.

## 5. Comparisons with Previous Models

**Figure 8:**
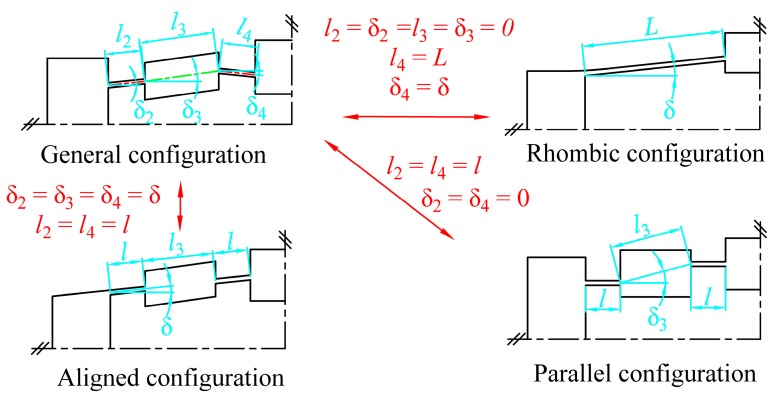
Variations of compliant bridge mechanisms between general, rhombic, parallel and aligned type configurations.

As shown in Figure 8, a general compliant bridge mechanism can be transformed into parallel, rhombic or aligned-type configurations by varying the six configuration parameters. By substituting the geometric characteristics of each configuration into the analytical equations, comparisons with previously developed models from the literature were carried out to investigate the feasibility of the models. 

First, a parallel configuration can be represented within the general framework by:

(22) $$\{\begin{array}{c} l_{2}=l_{4}=l\\ \delta_{2}=\delta_{4}=0 \end{array}$$

By substituting these configuration parameters into Equation (9), the general equation for displacement amplification can be written as:

(23) $$da_{\mathrm{parallel}}=\frac{\sin\delta_{3}\cdot \left(c_{33}^{l}\cdot \cos\delta_{3}\cdot l_{3}^{2}+2\cdot c_{23}^{l}\cdot l_{3}\right)}{c_{33}^{l}\cdot l_{3}^{2}\cdot \cos^{2}\delta_{3}+4\cdot c_{11}^{l}}$$

where $\;c_{ij}^{l}$ is the compliance factor of the flexure hinge corresponding to length l. Equation (23) is the same as that presented by Qi or Ling [23,26]. Secondly, the configuration parameters of the rhombic type compliant bridge mechanisms can be given as:

(24) $$\{\begin{array}{c} l_{2}=l_{3}=\delta_{2}=\delta_{3}=0\\ l_{4}=L\\ \delta_{4}=\delta \end{array}$$

By substituting the configuration parameters into Equation (9), the general equation of displacement amplification turns into:

(25) $$da_{\mathrm{rhombic}}=\frac{\sin\left(2\cdot \delta \right)\cdot \left(2\cdot c_{11}^{L}-\frac{c_{22}^{L}}{2}\right)}{c_{22}^{L}+4\cdot c_{11}^{L}\cdot \cos^{2}\delta -c_{22}^{L}\cdot \cos^{2}\delta }$$

where $\;c_{ij}^{L}$ is the compliance factor of the flexure hinge corresponding to length L. Equation (25) is the same as that presented by Ling [26] ( note that $c_{33}^{L}=\frac{3\cdot c_{22}^{L}}{L^{2}}$ and $c_{23}^{L}=c_{32}^{L}=\frac{6\cdot c_{22}^{L}}{4\cdot L}$ have been applied as indicated in Equation (2) for strip type flexure hinges). Hence, it can be concluded that the presented models generalize both the parallel and rhombic type compliant bridge mechanism models that have been verified by previous studies. However, the equation for displacement amplification of the aligned-type compliant bridge mechanisms has not yet been investigated. The configuration parameters of the aligned-type compliant bridge mechanisms can be described as:

(26) $$\{\begin{array}{c} \delta_{2}=\delta_{3}=\delta_{4}=\delta \\ l_{2}=l_{4}=l \end{array}$$

By substituting the configuration parameters into Equation (9), the equation for displacement amplification of aligned-type mechanisms is determined to be:

(27) $$da_{\mathrm{aligned}}=\frac{\sin\delta \cdot \cos\delta \cdot \left(4\cdot c_{22}^{l}-4\cdot c_{11}^{l}+2\cdot c_{23}^{l}\cdot l_{3}+2\cdot c_{32}^{l}\cdot l_{3}+c_{33}^{l}\cdot l_{3}^{2}\right)}{4\cdot c_{22}^{l}\cdot \sin^{2}\delta +4\cdot c_{11}^{l}\cdot \cos^{2}\delta +2\cdot l_{3}\cdot c_{23}^{l}\cdot \sin^{2}\delta +2\cdot l_{3}\cdot c_{32}^{l}\cdot \sin^{2}\delta +l_{3}^{2}\cdot c_{33}^{l}\cdot \sin^{2}\delta }$$

**Figure 9:**
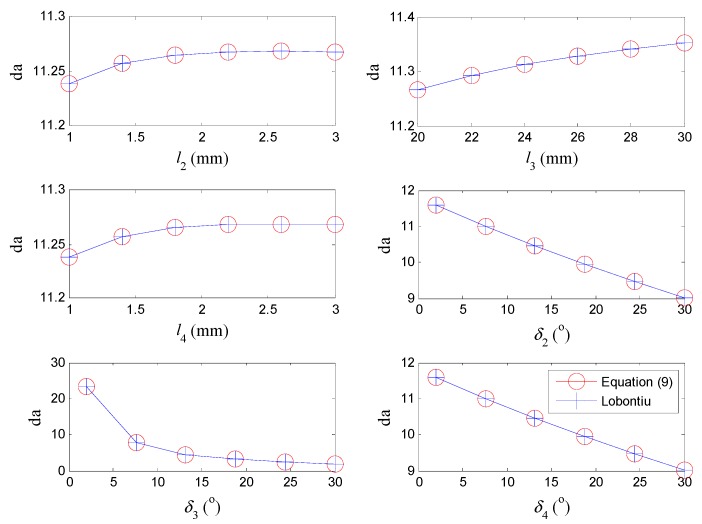
Numerical comparisons of displacement amplification between the new and Lobontiu’s equations in terms of the configuration parameters.

Furthermore, numerical simulations were carried out to compare the presented equations with those proposed by Lobontiu [18] in terms of the six configuration parameters for general complaint bridge mechanisms. During the computations, only one parameter is varied in each analysis, while the other parameters were kept constant, as: $l_{2}=l_{4}=0.002\;m$, $l_{3}=0.02\;m$, $\delta_{2}=\delta_{3}=\delta_{4}=5{}^{\circ}$. The thickness and width of the flexure hinge are fixed at: $t_{2}=t_{4}=0.0004\;m$, w=0.004 m. As shown in Figure 9, the results calculated by the proposed equations match well with those obtained by Lobontiu’s equations This suggests that the presented models are feasible for compliant bridge mechanisms in general configurations for both macro and micro applications. 

## 6. Conclusions

In this study, a simplified analytical model for general compliant bridge mechanisms has been formulated based on beam theory and kinematic analysis. The model has been shown to accurately characterize compliant bridge mechanisms in parallel, aligned and rhombic type configurations. Analytical equations of input, output, displacement amplification, stiffness and stress have been obtained. The optimization of a piezo-driven compliant bridge mechanism has been accomplished based on the proposed models and equations. With the presented equations, optimizations can be achieved efficiently. The aligned configuration was found to be globally optimal within this framework. The optimal design was developed and investigated by FEA and experiment. The deviations between analytical displacement amplification and FEA and experiment are less than 8% and 4%, respectively. Comparisons with previous equations have indicated that the presented models are feasible for general compliant bridge mechanisms for both macro and micro applications. The equation for displacement amplification for aligned-type compliant bridge mechanisms was first obtained. The concise form of the proposed equations can help to facilitate the optimal design of compliant bridge mechanisms. Future work will be directed toward the nonlinear modeling of large deformation or material nonlinearity, dynamic modeling and precision control of the compliant bridge mechanisms.

## Figures and Tables

**Table 1:** Boundary of the geometric parameter for optimization of the piezo-driven compliant bridge mechanism and the global optimal result.

Parameters (mm, °)	$l_{2}$	$\delta_{2}$	$t_{2}$	$l_{3}$	$\delta_{3}$	$l_{4}$	$\delta_{4}$	$t_{4}$
Upper boundary	20	45	2	20	45	20	45	2
Lower boundary	0.5	−45	0.4	0.5	−45	0.5	−45	0.4
Optimal result	1.96	4.07	0.4	8.1	4.01	1.96	4.07	0.4

**Table 2:** Performance of the global optimal compliant bridge mechanism by finite element analysis (FEA) and analytical equations.

Result	$Y_{\mathrm{out}}$ (µm)	$X_{\mathrm{in}}$ (µm)	$K_{\mathrm{in}}$ (N/m)	$\sigma^{\max}$ (MPa)	$K_{\mathrm{out}}$ (N/m)
FEA	18.6	1.56	6.43 × 10^6^	165	3.9 × 10^4^
Analytical	18.3	1.42	7.02 × 10^6^	148	3.8 × 10^4^
Deviation	1.5%	8.5%	9.2%	10.3%	2.5%

**Table 3:** Analytical, FEA and experimental results of displacement amplification for the developed compliant bridge mechanism.

Types of Result	FEA	Experimental	Analytical
da	11.95	12.44	12.86

## Appendix A

The coefficients in Equations (8)−(11) are given as:

(A1) $$\begin{array}{cc} a_{11}= & \sin\delta_{2}\left(c_{22}^{b}\cdot \sin\delta_{2}+\frac{c_{23}^{b}\cdot \left(c_{32}^{d}\cdot \sin\delta_{4}-c_{32}^{b}\cdot \sin\delta_{2}+c_{33}^{d}\cdot l_{3}\cdot \sin\delta_{3}\right)}{c_{33}^{b}+c_{33}^{d}}\right)\\ & \;+\sin\delta_{4}\left(c_{22}^{d}\cdot \sin\delta_{4}+\frac{c_{23}^{d}\cdot \left(c_{32}^{b}\cdot \sin\delta_{2}-c_{32}^{d}\cdot \sin\delta_{4}+c_{33}^{b}\cdot l_{3}\cdot \sin\delta_{3}\right)}{c_{33}^{b}+c_{33}^{d}}\right)\\ & \;+c_{11}^{b}\cdot \cos^{2}\delta_{2}+c_{11}^{d}\cdot \cos^{2}\delta_{4}\\ & \;+\frac{l_{3}\cdot \sin\delta_{3}\cdot \left(c_{32}^{b}\cdot c_{33}^{d}\cdot \sin\delta_{2}+c_{32}^{d}\cdot c_{33}^{b}\cdot \sin\delta_{4}+c_{33}^{b}\cdot c_{33}^{d}\cdot l_{3}\cdot \sin\delta_{3}\right)}{c_{33}^{b}+c_{33}^{d}} \end{array}$$

(A2) $$\begin{array}{cc} a_{12}= & c_{11}^{b}\cdot \cos\delta_{2}\cdot \sin\delta_{2}\\ & \;-\sin\delta_{4}\left(c_{22}^{d}\cdot \sin\delta_{4}+\frac{c_{23}^{d}\cdot \left(c_{32}^{b}\cdot \cos\delta_{2}-c_{32}^{d}\cdot \cos\delta_{4}+c_{33}^{b}\cdot l_{3}\cdot \cos\delta_{3}\right)}{c_{33}^{b}+c_{33}^{d}}\right)\\ & \;-\sin\delta_{2}\left(c_{22}^{b}\cdot \sin\delta_{2}+\frac{c_{23}^{b}\cdot \left(c_{32}^{d}\cdot \cos\delta_{4}-c_{32}^{b}\cdot \cos\delta_{2}+c_{33}^{d}\cdot l_{3}\cdot \cos\delta_{3}\right)}{c_{33}^{b}+c_{33}^{d}}\right)\\ & \;+c_{11}^{d}\cdot \cos\delta_{4}\cdot \sin\delta_{4}\\ & \;-\frac{l_{3}\cdot \sin\delta_{3}\cdot \left(c_{32}^{b}\cdot c_{33}^{d}\cdot \cos\delta_{2}+c_{32}^{d}\cdot c_{33}^{b}\cdot \cos\delta_{4}+c_{33}^{b}\cdot c_{33}^{d}\cdot l_{3}\cdot \cos\delta_{3}\right)}{c_{33}^{b}+c_{33}^{d}} \end{array}$$

(A3) $$\begin{array}{cc} a_{21}= & \cos\delta_{2}\left(c_{22}^{b}\cdot \sin\delta_{2}+\frac{c_{23}^{b}\cdot \left(c_{32}^{d}\cdot \sin\delta_{4}-c_{32}^{b}\cdot \sin\delta_{2}+c_{33}^{d}\cdot l_{3}\cdot \sin\delta_{3}\right)}{c_{33}^{b}+c_{33}^{d}}\right)\\ & \;+\cos\delta_{4}\left(c_{22}^{d}\cdot \sin\delta_{4}+\frac{c_{23}^{d}\cdot \left(c_{32}^{b}\cdot \sin\delta_{2}-c_{32}^{d}\cdot \sin\delta_{4}+c_{33}^{b}\cdot l_{3}\cdot \sin\delta_{3}\right)}{c_{33}^{b}+c_{33}^{d}}\right)\\ & \;-c_{11}^{b}\cdot \cos\delta_{2}\cdot \sin\delta_{2}-c_{11}^{d}\cdot \cos\delta_{4}\cdot \sin\delta_{4}\\ & \;+\frac{l_{3}\cdot \sin\delta_{3}\cdot \left(c_{32}^{b}\cdot c_{33}^{d}\cdot \sin\delta_{2}+c_{32}^{d}\cdot c_{33}^{b}\cdot \sin\delta_{4}+c_{33}^{b}\cdot c_{33}^{d}\cdot l_{3}\cdot \sin\delta_{3}\right)}{c_{33}^{b}+c_{33}^{d}} \end{array}$$

(A4) $$\begin{array}{cc} a_{22}= & -\frac{l_{3}\cdot \cos\delta_{3}\cdot \left(c_{32}^{b}\cdot c_{33}^{d}\cdot \cos\delta_{2}+c_{32}^{d}\cdot c_{33}^{b}\cdot \cos\delta_{4}+c_{33}^{b}\cdot c_{33}^{d}\cdot l_{3}\cdot \cos\delta_{3}\right)}{c_{33}^{b}+c_{33}^{d}}-c_{11}^{b}\cdot \sin^{2}\delta_{2}\\ & \;-c_{11}^{d}\cdot \sin^{2}\delta_{4}\\ & \;-\cos\delta_{2}\left(c_{22}^{b}\cdot \cos\delta_{2}+\frac{c_{23}^{b}\cdot \left(c_{32}^{d}\cdot \cos\delta_{4}-c_{32}^{2}\cdot \cos\delta_{2}+c_{33}^{d}\cdot l_{3}\cdot \cos\delta_{3}\right)}{c_{33}^{b}+c_{33}^{d}}\right)\\ & \;-\cos\delta_{4}\left(c_{22}^{d}\cdot \cos\delta_{4}+\frac{c_{23}^{d}\cdot \left(c_{32}^{b}\cdot \cos\delta_{2}-c_{32}^{d}\cdot \cos\delta_{4}+c_{33}^{b}\cdot l_{3}\cdot \cos\delta_{3}\right)}{c_{33}^{b}+c_{33}^{d}}\right) \end{array}$$
